# Exercise stress echocardiography shows impaired left ventricular function after hospitalization with COVID‐19 without overt myocarditis: A pilot study

**DOI:** 10.14814/phy2.70138

**Published:** 2024-11-28

**Authors:** Robert E. Goldstein, Edward A. Hulten, Thomas B. Arnold, Victoria M. Thomas, Andrew Heroy, Erika N. Walker, Keiko Fox, Hyun Lee, Joya Libbus, Bethelhem Markos, Maureen N. Hood, Travis E. Harrell, Mark C. Haigney

**Affiliations:** ^1^ Military Cardiovascular Outcomes Research Program Uniformed Services University of the Health Sciences Bethesda Maryland USA; ^2^ Walter Reed National Military Medical Center Bethesda Maryland USA; ^3^ Henry M. Jackson Foundation Bethesda Maryland USA; ^4^ Metis Foundation San Antonio Texas USA; ^5^ Department of Radiology Uniformed Services University of the Health Sciences Bethesda Maryland USA; ^6^ Present address: Department of Medicine Alpert Medical School of Brown University Providence Rhode Island USA

**Keywords:** COVID‐19, left ventricular dysfunction, stress echocardiography

## Abstract

Usual clinical testing rarely reveals cardiac abnormalities persisting after hospitalization for COVID. Such testing may overlook residual changes causing increased adverse cardiac events post‐discharge. To clarify status post‐hospitalization, we related exercise stress echocardiography (ESE) in 15 recovering patients (RP) age 30–63 without myocarditis to matching published data from healthy subjects (HS). RP exercise, average duration 8.2 ± 2.2 SD, was halted by dyspnea or fatigue. RP baselines matched HS except for higher heart rate. At peak stress, RP had significantly lower mean left ventricular (LV) ejection fraction (67% ± 7 vs. 73% ± 5, *p* < 0.0017) and higher peak early mitral inflow velocity/early mitral annular velocity (E/e’, 9.1 ± 2.5 vs. 6.6 ± 2.5, *p* < 0.006) compared with HS performing equal exercise (8.5 ± 2.6 min). Thus, when stressed, patients without known cardiac impairment showed diminished systolic contractile function and diastolic LV compliance vs. HS. RP peak heart rate was significantly higher (172 ± 18 vs. 153 ± 20); peak systolic blood pressure trended higher (192 ± 31 vs. 178 ± 19). Pulmonary artery systolic pressures among RP remained normal. ESE uniquely identified residual abnormality in cardiac contractile function not evident unstressed, exposing previously unrecognized residual influence of COVID‐19. This may reflect autonomic dysfunction, microvascular disease, or diffuse interstitial changes; these results may have implications for clinical management and later prognosis.

## Graphical abstract

Cardiac impairment at peak stress after hospitalization for COVID‐19 without overt myocarditis

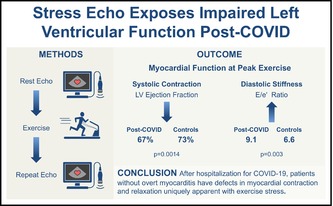

## 1 INTRODUCTION

COVID‐19 can adversely affect the heart and circulation, particularly in hospitalized patients (Raisi‐Estabragh et al., 2022). Early experience demonstrated that severe acute COVID‐19 infection can lead to heart failure and arrhythmia (Nishiga et al., 2020). Cardiovascular abnormalities may persist into the recovery period and contribute to disability lasting many months. Recent studies have shown substantially increased occurrence of multiple different adverse clinical cardiovascular consequences in the year after hospitalization for COVID‐19 compared with COVID‐infected but non‐hospitalized patients and compared with uninfected patients (Bowe et al., 2023; Hilser et al., 2024; Xie et al., 2022). While lingering direct viral or immune actions may play a role, the reasons for these persistent cardiovascular problems and their long‐term implications remain unclear.

Routine testing of blood troponin, brain natriuretic peptide, and C‐reactive protein shows no abnormalities during recovery in nearly all patients hospitalized with COVID‐19 without overt myocarditis during or after their hospital stay (Nalbandian et al., 2023; Richard et al., 2023). The same lack of abnormality is also observed when these patients have electrocardiograms, echocardiograms, or magnetic resonance images (Joy et al., 2021; Udelson et al., 2021; Writing Committee for the COMEBAC Study Group et al., 2021). However, prior investigations have not assessed the performance of the heart and circulation during maximal exercise‐induced stress, which may bring out abnormalities that are inapparent in the absence of such stress. Treadmill performance limited by cardiovascular symptoms such as fatigue or dyspnea is likely to elicit maximal cardiac function, as evidenced by substantially increased left ventricular ejection fraction (LVEF) and stable diastolic performance parameters (Ha et al., 2003). Sensitive detection of cardiovascular abnormalities after hospitalization with COVID‐19 may have value in identifying those at risk for later complications. In addition, assessment of exercise stress performance may have benefit in choosing proper activities for recovering patients. Accordingly, we evaluated treadmill echocardiographic performance at least 6 weeks after hospital discharge using standard clinical methods in patients without evidence of myocarditis during or after hospitalization for COVID‐19.

Utilizing exercise stress echocardiography (ESE), we assessed whether cardiac contractile performance at symptom‐limited maximum effort among recovering patients (RP) after hospitalization with COVID‐19 would differ from published data (Ha et al., 2003) for similarly elicited ESE results in healthy comparison subjects (HS) with no exposure to COVID‐19. Our pre‐specified primary hypothesis was that RP and HS would show no statistically significant difference in LVEF soon after maximum stress. Our secondary hypothesis was that measurements made during the same maximal stress assessment would demonstrate no statistically significant difference in early diastolic stiffness (noncompliance), measured as peak early mitral inflow velocity (E) over the early diastolic mitral annular velocity (e’), E/e’, between RP and HS.

## 2 MATERIALS AND METHODS

### 2.1 Patients previously hospitalized with COVID‐19

To recruit recovering patients, advertisements were posted in appropriate outpatient clinics at Walter Reed National Military Medical Center, and referrals were solicited from staff physicians who managed care for patients admitted to the hospital with COVID‐19. All patients eligible for military health care and age <65 years without prior cardiovascular diagnosis other than essential hypertension were eligible for study enrollment >6 weeks post‐discharge from the hospital after admission with a positive PCR test for COVID‐19. Each study enrollee had an interview and an ECG and serum troponin, brain natriuretic peptide, and d‐dimer levels at enrollment.

### 2.2 Healthy comparison subjects

During the COVID‐19 pandemic, limitation of patient resources and staff in our hospital precluded echocardiographic assessment in healthy comparison subjects without prior COVID‐19 infection. Data for comparison with performance of RP were selected from published literature based on demographic similarity of participants to our study patients except for COVID‐19 infection and an equivalent ESE protocol performed by a trusted clinical echocardiography unit (Ha et al., 2003). Prior to the COVID‐19 pandemic, 31 HS were assessed according to the same ESE protocol used in the present study. Their performance is taken as comparison data for left ventricular function during exercise in our patients studied after hospitalization with COVID‐19. The Mayo Clinic developed these data specifically to provide baseline echocardiographic measures of cardiac performance in normal subjects during symptom‐limited exercise for comparison with outcomes in later studies of heart patients (Ha et al., 2003).

### 2.3 Exercise stress echocardiographic tests

Of our 20 enrolled patients, 15 were deemed appropriate for ESE based on (1) lack of evidence of myocarditis on current testing (ECG and troponin levels) or prior in‐hospital results (ECG, troponin, or MRI) indicative of myocarditis and (2) patient‐observed ability to walk a mile or climb 3 flights at a normal pace without pause at the time of study enrollment. All ESE testing was supervised and interpreted by experienced, echo board‐certified cardiologists (E.H. and T.H.) and other experienced clinical personnel according to a pre‐specified protocol used for routine clinical ESE that included baseline blood pressure, SpO2, ECG, and cardiac ultrasound measurements. Patients then exercised on a motor‐driven treadmill according to the standard Bruce protocol with continuous ECG monitoring and cuff measurement of blood pressure every 3 minutes and just before stopping.

Exercise was continued until halted by symptoms judged to preclude further effort. The final echocardiogram was recorded within 2 min of stopping exercise.

### 2.4 Analysis of echocardiograms

Imaging and Doppler echocardiograms were recorded using a single General Electric Vivid E9 unit. LVEF was calculated from the 2‐dimensional echocardiography data using the American Society of Echocardiography recommended technique, a biplane method of disks summation (modified Simpson's rule). For diastolic evaluation using an apical window, the pulsed Doppler sample volume was placed at the mitral valve tips, and 5–10 cardiac cycles were recorded and averaged. Mitral inflow velocities were recorded, and the peak velocity of early filling (E) was derived. Special pulse‐wave Doppler mode was used to assess tissue velocities. For measurements of mitral annular velocities (e’), the filter was set to exclude high‐frequency signals and Nyquist limit adjusted to 15–20 cm/s. Gain was reduced to obtain a clear tissue signal with minimal background noise. From the apical 4‐chamber view, a 2‐ to 5‐mm sample volume was placed at the septal corner of the mitral annulus. The E/e’ ratio was calculated from paired mean values of E and e’. Maximal tricuspid regurgitation velocity (TRV_max_) was evaluated in apical and subcostal views; for each patient, TRV_max_ was averaged over a single respiratory cycle. Right atrial pressure was estimated using inferior vena cava size and inspiratory collapse in subcostal view (Lang et al., 2015; Rudski et al., 2010). Pulmonary artery systolic pressure was calculated as 4 (TRV_max_)^2^ + estimated right atrial pressure, per usual standard practice. The measurements just described were obtained in the same sequence before and after exercise.

The echocardiographic measurement techniques and analytic methods just presented for RP were the same as those described for the Mayo Clinic assessment of HS (Ha et al., 2003).

### 2.5 Statistics

Statistical methods were selected to investigate potential associations between clinical parameters and ESE results in RP as well as compare hemodynamic performance in RP and HS. After accounting for missing data, the RP sample size was deemed insufficient to support multivariate analysis. We performed univariate analyses, which, despite limitations, allowed examination of individual relationships between prespecified variables and primary ESE outcomes. Univariate correlations and significance between individual variables and the primary ESE outcomes were calculated. Mean values are shown ± standard deviation. After confirming the normality of comparator variables within the RP sample (LVEF at rest, stress LVEF, E/e’ at rest, and stress E/e’) using the Shapiro–Wilk test, corresponding mean values for RP and HS were compared using the unpaired Student's *t*‐test, deemed valid based on similar sample sizes and variances for comparator variables. Values for all statistical tests were deemed statistically significant if *p* < 0.05. A larger and more diverse sample is required to fully investigate the interplay of factors potentially involved in measured responses; the limited sample size precluded linear modeling.

### 2.6 Protocol approval

The protocol for this study (WRNMMC‐2020‐0319) received final approval from the Walter Reed Investigational Review Board on Dec 1, 2020. Patient enrollment occurred May 10, 2021‐June 30, 2022.

## 3 RESULTS

### 3.1 Patient characteristics

Recovering patients (RP) after COVID‐19 were age 49 ± 11 years; age range was 30–63 years (Table 1). Nine (60%) were women and six were men. Seven (47%) were active‐duty service members. Body mass index averaged 33.7 ± 6.2 kg/m^2^; eight patients were >30 kg/m^2^. Eight (53%) were on antihypertensive medications, including 3 on beta‐blocking drugs (dose held the morning of ESE). One patient, a woman in her 40's, took medications to control diabetes. This patient had no known prior cardiovascular disorder besides mild, uncomplicated essential hypertension.

**TABLE 1:** Study Subjects Having Exercise Stress Echocardiography (*n* = 15).

*Demographics*	
Age (Years)	
Mean ± Std. Dev	49.1 ± 10.6
Sex	*N* (%)
Male/Female	6 (40%)/9 (60%)
Active duty	*N* (%)
Yes/No	7 (46.7%)/8 (53.3%)
Body Mass Index (kg/m^2^)	
Mean ± Std. Dev	33.7 ± 6.2
*Hospital Course*	
Length of stay (Days)	
Mean ± Std. Dev	6.7 ± 3.6
ICU Admission	*N* (%)
Yes/No	8 (53.3%)/7 (46.7%)
COVID pneumonia and/or acute respiratory failure	*N* (%)
Yes/No	11 (73.3%)/4 (26.7%)
Days from hospital discharge to ESE	
Mean ± Std. Dev	448 ± 258
*Current Medications at ESE*	
Antihypertensives	*N* (%)
Yes/No	8 (53.3%)/7 (46.7%)
Beta blockers	*N* (%)
Yes/No	3 (20%)/12 (80%)
Diabetes medications	*N* (%)
Yes/No	1 (6.7%)/14 (93.3%)

Abbreviation: ESE, exercise stress echocardiogram.

RP were admitted to hospital between April 2020 and January 2022; each had a positive PCR test for COVID‐19 when hospitalized. Twelve (80%) had no prior covid‐19 vaccination, and none had known prior infection with COVID‐19. Eight (53%) were treated in the intensive care unit. Total hospital stay averaged 6.7 ± 3.6 days. Principal discharge diagnosis was COVID‐19 pneumonia in 10 (67%) with accompanying acute hypoxic respiratory insufficiency in 4. Other individual patients were admitted for pericarditis without myocarditis, branch‐vessel pulmonary embolism, neurological symptoms, pancreatitis, and severe emesis. While in hospital, 4 patients received dexamethasone; additionally, 3 had remdesivir (2 with dexamethasone), 2 had convalescent plasma, and one had tocilizumab (each with dexamethasone). Ten patients had only conventional treatment for viral pneumonia.

Prior to enrollment, all patients provided written informed consent. At study enrollment, all 15 having ESE had a normal electrocardiogram and normal values for serum high‐sensitivity troponin T, brain natriuretic peptide, and d‐dimer.

Historically‐derived healthy comparison subjects (HS) were age 59 ± 14 years, and 18 of the 31 (58%) were women. Except for prior COVID‐19 exposure, subjects generally resembled RP: they were middle‐aged, without manifestations of cardiovascular disorder, and capable of moderate exercise but were not known athletes. However, their average age was 10 years older than RP. HS also differed from RP in that none had hypertension and none took medications.

### 3.2 Exercise stress echocardiographic test outcomes

ESE testing was performed 448 ± 258 days after hospital discharge (range 97–949 days). All patients wore face masks at rest and during exercise, in keeping with hospital safety regulations. At the outset, all 15 patients had normal (>95%) SpO2.

Patients walked on a treadmill at increasing speeds and grades following the standard Bruce protocol until halted by symptoms. During exercise, patients were queried every 3 min about the quality and intensity of symptoms, referring to a posted Borg Scale. In the final stages, each patient rated the effort “hard” or “very hard.” These symptoms included limiting fatigue and dyspnea in each; some subjects had accompanying leg pain or pleuritic‐type chest pain. None had ischemic‐type chest pain. All patients remained in sinus rhythm during exercise and recovery; none had associated abnormal ST‐T wave changes. In response to detailed patient coaching and in accord with extensive staff practice, all study patients had post‐exercise echocardiograms recorded 1–2 min after halting exercise. HS performed treadmill exercise according to the same Bruce protocol and were limited by fatigue or dyspnea. Echo methods of recording and interpretation for HS were the same as those used for RP (Ha et al., 2003).

**FIGURE 1:**
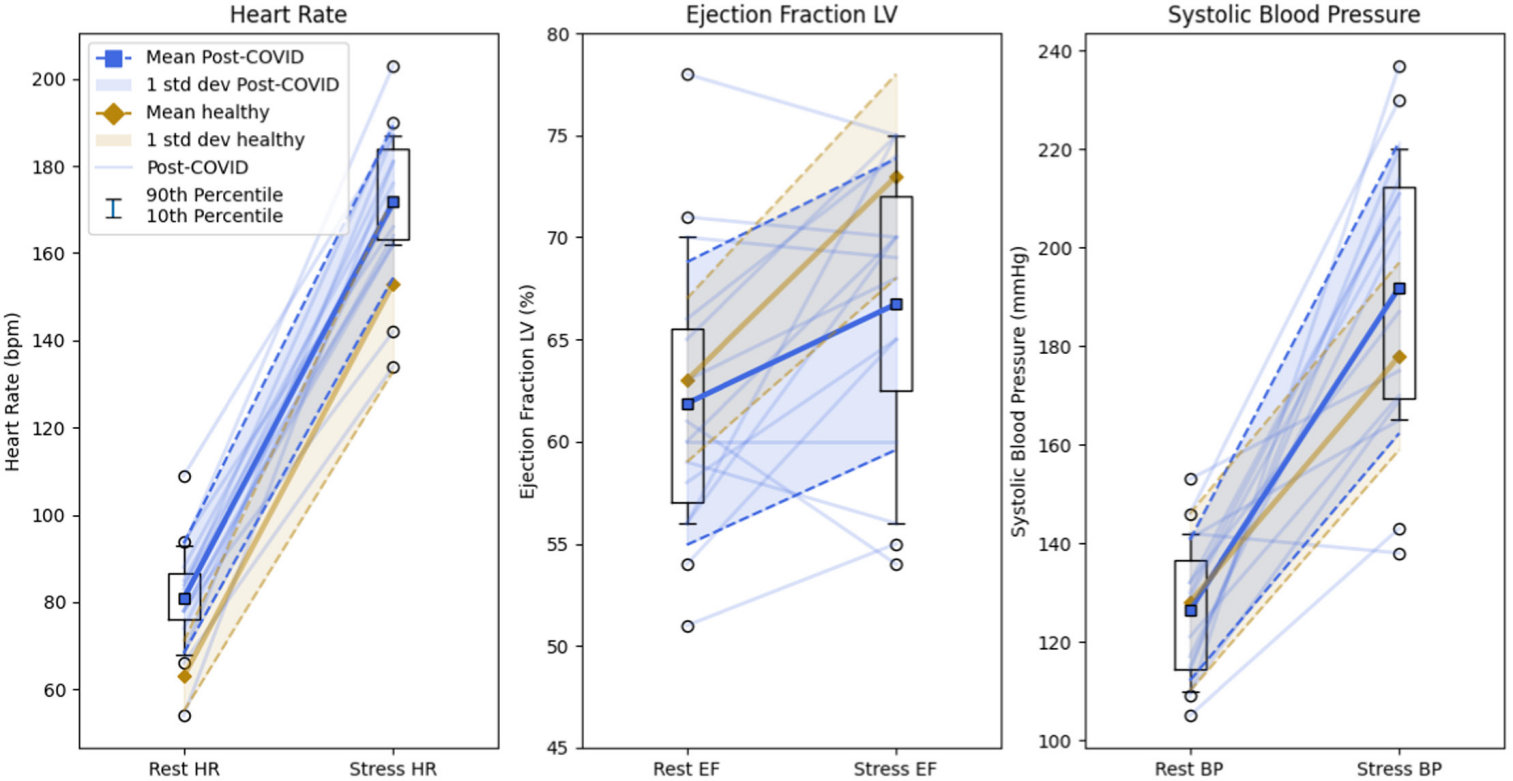
In each panel, values at supine rest pre‐stress (left) are connected to corresponding values at peak symptom‐limited treadmill stress (right). It depicts heart rate (left panel), left ventricular ejection fraction (LVEF, middle panel), and systolic blood pressure (BP, right panel). Data for the 15 recovering patients (RP) are shown in blue, and data for 31 healthy comparison subjects (HS) in gold. Means are represented by squares/diamonds and thick lines; one standard deviation is shown as shaded areas bounded by dashed lines. For RP data, “box and whisker” plots denote the 25th and 75th percentiles (bottom and top of boxes) plus 10th and 90th percentiles (whiskers). “Fliers” outside those whiskers are shown as circles. Individual performance for RP is indicated by fine lines; individual performance for HS is not available. Mean stress LVEF for RP is significantly below stress LVEF for HS. Heart rate is also significantly higher at rest and peak stress. Exercise BP tends higher in RP but the difference from HS is not statistically different (*p* = 0.0647).

At supine rest before exercise, RP had hemodynamic parameters the same as HS, except for a higher mean heart rate (Table 2, upper panel and Figure 1). Specifically, LVEF and E/e’ were not different from HS. Pulmonary artery systolic pressure, estimated at rest in 8 RP, was normal (<36 mmHg) (Ha et al., 2003) in each individual (group mean 25 ± 6 mmHg). Regional wall motion for RP, assessed in each of the 15, was normal at rest and after treadmill stress, and no chamber enlargement was observed.

**TABLE 2:** Cardiac Parameters at Pre‐Stress Rest and Peak Treadmill Exercise.

	Ex duration (min)	LVEF (%)	E/e’	SBP (mmHg)	HR (bpm)
Rest performance					
Post‐COVID	N/A	62 ± 7	7.7 ± 3.7	126 ± 15	81 ± 13†
Healthy Subjects	N/A	63 ± 4	6.7 ± 2.2	128 ± 18	63 ± 8
Stress performance					
Post‐COVID	8.2 ± 2.2	67 ± 7‡	9.1 ± 2.5*	192 ± 31	172 ± 18◊
Healthy Subjects	8.5 ± 2.6	73 ± 5	6.6 ± 2.5	178 ± 19	153 ± 20

*Note*: Compared with Healthy Subjects: †*p* < 0.0001; ‡*p* = 0.0014; *p = 0.0031; ◊*p* = 0.0032.

Abbreviations: E/e’, peak early mitral inflow velocity (E) over the early diastolic mitral annular velocity (e’); HR, heart rate; Ex, exercise; LVEF, left ventricular ejection fraction; SBP, systolic blood pressure.

RP performed according to the same exercise protocol as HS and stopped due to limiting fatigue or dyspnea after mean 8.2 ± 2.2 min, nearly the same as the symptomatic stopping time for HS (Table 2, lower panel). By contrast, mean final LVEF for RP was significantly lower (66.7 ± 7.4% RP vs. 73 ± 5% HS, *p* = 0.0014) (Figure 1, middle panel and Table 2) and mean E/e’ (*n* = 14) significantly higher (9.1 ± 2.5 RP vs. 6.6 ± 2.5 HS, *p* = 0.0031). In addition, mean peak heart rate was significantly higher (172 ± 18 bpm RP vs. 153 ± 20 HS, *p* = 0.0031) (Figure 1, left panel and Table 2). RP reached 100.3% ±7.7 of maximum predicted heart rate (MPHR); mean MPHR for HS was 95%. Systolic blood pressure values at end‐exercise tended higher in RP compared with HS but were not consistently so (*p* = 0.0647) (Figure 1, right panel). Among study subjects, average pulmonary artery systolic pressure for RP (*n* = 13) at end‐exercise was 31 ± 9 mmHg. In 6 RP with paired estimates, mean rise in pulmonary artery systolic pressure was 5.7 ± 1.7 mmHg (*p* = 0.023).

Correlation coefficients and their associated p‐values were calculated for RP (Table 3). This correlation analysis identified no association of stress LVEF in study patients with body mass index, prior use of antihypertensive medication, age, gender, or systolic blood pressure. The correlation analysis also indicated no association of stress E/e’ with these same covariates.

**TABLE 3:** Correlation Matrix of Selected Patient Variables.

	Age	Gender	BMI	Anti‐HTN Med	Stress Systolic BP	Stress LVEF	Stress E/e’
Age	1						
Gender	0.018	1					
BMI	0.277	−0.134	1				
Anti‐HTN Med	**0.600**ᵻ	0.043	0.573*	1			
Stress systolic BP	−0.109	−0.013	0.031	−0.124	1		
Stress LVEF	0.137	−0.382	0.043	−0.247	−0.101	1	
Stress E/e’	0.227	−0.09	0.219	0.32	−0.22	−0.19	1

*Note*: Correlation matrix assesses the linkage of 7 prespecified clinical characteristics and hemodynamic values at exercise stress echocardiography for the 15 patients recovering after hospitalization for COVID‐19. Numerical values ranging from −1 to 1 indicate the spearman correlation, or strength, of the paired linkage between horizontally and vertically arrayed features (identity = 1). Antihypertensive therapy was significantly correlated with age and body mass index. However, Stress LVEF and E/e’ were not correlated with any of the clinical parameters (*p* > 0.05 for each). ᵻ*p* = 0.0233, *p = 0.0322.

Abbreviations: Anti‐HTN Med, antihypertensive medications; BMI, body mass index; BP, blood pressure; E/e’, peak early mitral inflow velocity divided by early mitral annular velocity; LVEF, left ventricular ejection fraction.

### 3.3 Clinical follow‐up

All study patients had 6‐monthly structured telephone interviews to assess functional capacity and clinical occurrences. Each of the 15 with ESE was followed for at least a year after study enrollment. Elicited information covered 20.1 patient‐years. RP had no deaths, myocardial infarctions, or strokes and no symptoms suggesting heart failure or sustained arrhythmia. Two of the 15 with ESE were hospitalized, 1 patient twice for pneumonia. At the completion of follow‐up for these 15 RP (averaging 1.3 years), 7 were deemed to function in New York Heart Association Class 1 and 8 in NYHA Class 2.

## 4 DISCUSSION

Our 15 middle‐aged study patients recovering from severe COVID‐19 without overt myocarditis achieved the same duration of symptom‐limited treadmill exercise as comparable uninfected individuals. However, their cardiovascular parameters at this same peak exercise were distinctly different: recovering patients had mildly but significantly diminished immediate post‐exercise left ventricular performance, evident in both systolic contraction—lesser LVEF—and diastolic relaxation—increased E/e’. In addition, recovering patients had heart rates at peak exercise significantly higher than healthy comparison subjects. Patients' systolic blood pressure at peak exercise tended to be higher, but this difference was not consistent.

Studying patients 6 months after hospitalization with COVID‐19, (Fayol et al., 2021) reported increased E/e’ after mild exercise but only in patients experiencing “myocardial injury” when in hospital. While supporting exertion‐related left ventricular diastolic dysfunction after COVID‐19, these data revealed functional abnormality only in patients with prior myocarditis. By contrast, our study showed performance of intense, symptom‐limited exercise was associated with systolic and diastolic dysfunction in subjects without prior overt myocarditis. Our more demanding exercise protocol may have elicited abnormalities in hospitalized COVID‐19 patients without myocarditis that are revealed only by intense effort. Our finding has distinctive implications: if confirmed, contractile abnormality in patients hospitalized with COVID‐19 but without myocarditis suggests a lingering defect in myocardial function not attributable to prior overt myocarditis.

Subnormal left ventricular performance at peak treadmill exercise was the only sign of cardiovascular dysfunction evident in our recovering patients. Echo parameters at rest, troponin levels, and the electrocardiogram showed no distinctive abnormality. Notably, our findings uniquely identified consistent subtle abnormalities of contractile function after severe COVID‐19 even in patients with no evidence of myocarditis on routinely surveyed parameters. These functional abnormalities, seen only during treadmill stress, suggest exercise testing is appropriate for those without overt myocarditis in the months after hospitalization for severe COVID‐19 to identify those needing longer recovery before returning to physically demanding activity. Such testing may also aid in early identification of post‐hospital patients at greatest risk of late‐appearing COVID‐related cardiac problems (Bowe et al., 2023; Hilser et al., 2024; Xie et al., 2022).

Other investigators of contractile function after COVID‐19 have reported abnormalities of global longitudinal strain (GLS), measured at rest (Kersten et al., 2023; Schellenberg et al., 2023; Shimoni et al., 2021). Shimoni et al (Shimoni et al., 2021) showed mildly impaired right ventricular and left ventricular GLS in patients who described COVID‐19 infection when compared to GLS in patients with no history of overt COVID‐19 infection. Schellenberg et al (Schellenberg et al., 2023) reported slight improvement of GLS during recovery from symptoms of COVID‐19. While these studies favor a post‐COVID defect in contractility, the small changes observed and lack of demonstrable connection to hemodynamic function during exercise limit interpretation of their data.

### 4.1 Potential meaning of study findings

Our data permit a tentative assessment of possible causes for the observed abnormalities in cardiovascular function. While some of our patients were overweight or on treatment for hypertension, univariate correlation analysis indicated that neither these conditions nor age were strongly linked to the abnormalities observed in left ventricular function. Heart rates are often higher in patients symptomatic after COVID‐19 at supine and upright rest (Larsen et al., 2021), an effect that may be mediated in part by deconditioning. Blood pressure may also be increased late after COVID‐19 (Tobler et al., 2022). Our data may reflect greater adrenergic drive and lesser vagal tone in our patients. Such effects may figure in the augmentation of peak heart rates and blood pressures, possibly enhanced because our patients were wearing masks during exercise. However, mask‐related enhancement of heart rate and blood pressure with exercise appears minor (Bao et al., 2023). Some of the peak heart rate increase in RP may relate to their younger age, on average, 10 years less than HS.

Neither deconditioning nor increased adrenergic tone explains the changes we noted in systolic and diastolic left ventricular performance immediately after symptom‐limited exertion: published findings indicate maximal LVEF is not affected by training during maximal, symptom‐limited treadmill exercise (Rerych et al., 1980; Williams et al., 1984). Alternatively, the rises we observed in heart rate and blood pressure at maximal exercise may have been autonomically mediated responses to exercise‐induced myocardial dysfunction and resultant insufficient perfusion of exercising skeletal muscles.

Residual post‐COVID pulmonary vascular dysfunction might interfere with normal left ventricular performance by inducing right ventricular overload. This is unlikely since our patients had normal pulmonary artery pressures at rest and immediately after maximal exercise, in agreement with prior findings in COVID‐19 patients at hospital discharge (Baratto et al., 1985). Normal peak pulmonary artery pressures also mitigate against exercise‐induced hypoxia from residual lung abnormalities or mask‐wearing. All our patients had normal SpO2 values at rest before stress, and other investigators found that mask‐wearing causes only mild reductions in arterial oxygen content during exercise (Bao et al., 2023).

Myocardial ischemia might be a causal factor explaining our finding of impaired left ventricular performance during exercise following severe COVID. Coronary endothelial dysfunction (Charfeddine et al., 2021; Haffke et al., 2022) or diffuse interstitial changes in the myocardium after subclinical myocarditis may lead to insufficient rise in coronary blood flow during maximal exertion. Alternatively, persistent COVID‐19 interference with oxidative metabolism of cardiomyocytes might limit synthesis of high‐energy phosphate in recovering patients (Pozzi, 2022; Pozzi & Dowling, 2021), particularly during periods of high metabolic demand such as exercise. Neither the 12‐lead electrocardiograms monitoring our patients during exercise nor the patients' symptoms were indicative of myocardial ischemia. However, overt ischemic manifestations may not be apparent if the myocardial perfusion defect is modest or if impairment of oxidative metabolism in myocytes is mild.

### 4.2 Study limitations

Limitation of patient resources and staff in our hospital during the COVID‐19 pandemic constrained the number of RP in this study and precluded contemporary comparison measurements in healthy subjects. Although studied elsewhere, the subjects taken as a comparison group (HS) were demographically close to our recovering patients (RP).

A noteworthy dissimilarity between study groups is age: the average age for HS was 59 years and for RP 49 years. This is unlikely to account for the lesser LV performance of RP. On the contrary, an age‐related decrement in stressed cardiac performance among HS may have led to an underestimate of functional decline in RP.

Other dissimilarities are likely among certain subjects: 8 RP took antihypertensive medications, and 8 were obese (>30 kg/m^2^). No HS took medications; weight status among HS was not specifically addressed. Although our analysis indicated similar decrement in LV performance during maximal exertion among RP without antihypertensives or obesity, these potentially confounding factors must be examined in larger series to confirm a lack of influence.

The similar values for symptom‐limited exercise duration and similar echocardiographic findings at rest for HS and RP favor the comparability of groups and confirm that the same ESE techniques were employed for the current studies and for the historical comparisons. In addition, our selected comparison values for HS resemble peak stress LVEF measurements in younger normal subjects with different demographics (Studer Bruengger et al., 2014; Wang et al., 2021).

Statistical analysis was limited in our study because of the small number of subjects studied and the limited character of data available. Multivariate analysis was not feasible due to these small numbers, and univariate analysis—available for RP—had limited power to discern covariate influence also related to small numbers of subjects. Results in individual subjects were not available for HS, so univariate analysis could not be performed for this group.

Because of the small sample size and use of historical comparison subjects, our study findings are not definitive and should be interpreted with caution. Nevertheless, statistically significant, correlated differences emerged in systolic and diastolic left ventricular function specifically during exercise stress even with a modest number of RP studied under constraining circumstances.

Our study population may have experienced uniquely intense COVID‐19 infection and its sequelae at hospitalization due to pandemic conditions and, for 80% of our patients, lack of prior vaccination. While the worst features of the COVID‐19 pandemic have abated, the abnormality of cardiac function at peak exercise seen in our patients represents a previously unrecognized aspect of COVID‐19 infection, one that may have significant implications for select patients with ongoing COVID‐19 vulnerability and for patients encountering future coronavirus infections.

## AUTHOR CONTRIBUTIONS

R.G. and M.C.H. conceived and designed research; E.H., E.W., K.F., H.L., J.L., B.M., and T.H. performed experiments; E.H., T.A., and V.T. analyzed data; R.G., E.H., T.A., and M.C.H. interpreted results of experiments; A.H. prepared figures; R.G. drafted manuscript; M.C.H., E.H., T.A., M.N.H., and M.C.H. edited and revised manuscript; R.G., E.H., T.A., V.T., E.W., A.H., K.F., H.L., J.L., B.M., M.N.H., T.H., and M.C.H. approved final version of manuscript.

## FUNDING INFORMATION

Partial funding support for this study was received from Award # HU00012120008 from the Defense Health Agency to the Military Cardiovascular Outcomes Research program, Uniformed Services University of the Health Sciences, Bethesda, MD.

## CONFLICT OF INTEREST STATEMENT

No conflicts of interest, financial or otherwise, are declared by the authors.

## DISCLAIMERS

The views expressed in this manuscript are those of the authors and do not reflect the official policy of the Department of Army/Navy/Air Force, the Uniformed Services University of the Health Sciences, the Department of Defense, or U.S. Government or the policy of the Henry M. Jackson Foundation or the Metis Foundation. This work was prepared by military and civilian employees of the U.S. Government as part of their official duties and therefore is in the public domain and does not possess copyright protection (public domain information may be freely distributed and copied; however, as a courtesy, it is requested that the Uniformed Services University of the Health Sciences and the authors be given appropriate acknowledgement).

## ETHICS STATEMENT

The Investigational Review Board of the Walter Reed National Military Medical Center supplied final approval for this study’s protocol on December 1, 2020. In accord with this protocol, each study subject provided written informed consent prior to study participation.
